# The Impact of Higher BMI on Wound Complications Following Adolescent Breast Reduction: A Retrospective Study of 1215 Cases

**DOI:** 10.1007/s00266-024-04048-4

**Published:** 2024-05-08

**Authors:** Victor J. Yu, Jason T. Pham, Adam G. Evans, Yifan Guo

**Affiliations:** Division of Plastic and Reconstructive Surgery, Eastern Virginia Medical School, 301 Riverview Avenue, Norfolk, VA USA

**Keywords:** Plastic surgery, Breast surgery, Pediatric surgery, Breast reduction

## Abstract

**Background:**

Macromastia is a physically and psychologically distressing condition for adolescents. While reduction mammaplasty is often the best treatment, risk factors for adolescent wound complications remain unclear. This study aims to investigate the impact of obesity and other predictors of postoperative wound complications following adolescent reduction mammaplasty using a national database.

**Methods:**

The 2012–2019 National Surgical Quality Improvement Program Pediatric (NSQIP-P) databases were reviewed to identify primary reduction mammaplasty encounters. World Health Organization Body Mass Index (BMI), alongside patient and case characteristics, were assessed for association for 30-day wound disruption or surgical site complications. Statistical analyses were performed to identify independent predictors for complications and determine a potential BMI cutoff for risk stratification.

**Results:**

There were 1215 patients with an average age of 16.6 years. The average BMI was 30.7 kg/m^2^, and 593 (48.8%) patients were nonobese while 622 (51.2%) were obese. The incidence of complications was 5.27%. Independent predictors of complications included a BMI 35–39.9, BMI  > 40, and an American Society of Anesthesiologists (ASA) Classification  > 3. A receiver operating characteristic curve determined that a BMI of 34.6 can be a potential cutoff for increased complication risk.

**Conclusions:**

Higher obesity increases risk of wound complications; however, complication rates remain low. A BMI of 34.6 is a potential screening metric for counseling and monitoring patients. Reduction mammaplasty should remain a viable option as it can significantly improve quality of life.

**Level of Evidence III:**

This journal requires that authors assign a level of evidence to each article. For a full description of these Evidence-Based Medicine ratings, please refer to Table of Contents or the online Instructions to Authors www.springer.com/00266.

## Introduction

Macromastia is oftentimes a physically challenging and psychologically distressing condition for many adolescent patients [1]. Back, shoulder and neck pain, and general discomfort are common physical problems, while psychological issues include unwanted attention, introversion, and development of eating disorders [2–4]. These issues are often intertwined and magnify one another, which can create an especially upsetting daily life for adolescents, during what is already a sensitive period in their life.

Reduction mammaplasty has been shown to improve these physical and psychosocial problems and is often the best treatment in certain groups of patients [5]. In adult patients, obesity and higher body mass index (BMI) have been shown to be independent risk factors for wound complications, such as dehiscence or surgical site infection [6, 7]. However, there is a relative paucity in the literature surrounding obesity as a potential risk factor for complications in the adolescent population.

Currently, most studies directed at this question have been single-center retrospective studies [8–11]. There is a previous study using a national database examined 542 patients, showing increased risk for postoperative adverse events in obese versus nonobese adolescents [12]. However, with the continuous rise in the number of adolescent reduction mammaplasties performed, increased reports and case submissions to national-level databases can be leveraged to provide more accurate and updated information regarding which risk factors are the largest contributors to wound complications. The purpose of this study is to use a national database to provide an update on adolescent reduction mammaplasty, the incidence of wound complications, and to stratify classes of obesity as potential risk factors for these complications occurring within 30 days. We hypothesize that some previously understood patient-based physical factors seen in adults, including obesity, would also prove to be a risk factor in adolescents.

## Methods

### Data

This study utilized the 2012–2019 American College of Surgeons' National Surgical Quality Improvement Program Pediatric (ACS NSQIP-P) database, which is a risk-adjusted outcomes-based database which is primarily used in for surgical quality improvement projects. This database excludes patients 18 years of age, or older, which excludes 19-year-old patients who fall under the World Health Organization (WHO) definition of adolescence as the second decade of life (10–19 years of age). For each encounter in the database, there are 240 variables collected. These include demographics, preoperative risk factors, intraoperative variables, and 30-day postoperative morbidity and mortality.

Patients were selected from the 2012–2019 releases of the NSQIP-P database using Current Procedural Terminology (CPT) code 19318 (reduction mammaplasty). Patient demographics, medical background, and 30-day outcomes data were abstracted. Individual BMI was calculated from the weight and height of the patient. BMI was then stratified based on World Health Organization (WHO) obesity classification (Nonobese <30.0, Obese (Class I) 30.0–34.9, Obese (Class II) 35–39.9, or Obese (Class III) >40 kg/m^2^). Multiple individual postoperative wound disruption/dehiscence and surgical site infection complication variables were combined into a single complication variable as the primary outcome measure.

### Statistical Analysis

Univariate analysis was performed to compare patients without complications and those with complications by using Pearson Chi-squared or Fisher exact test for the categorical variables; continuous variables were examined using independent sample t-test or Mann–Whitney U tests. Cases with missing variables were not included in analyses. We aimed to be as inclusive as possible and fitted all relevant variables into a multivariable logistic regression with our composite complication variable as the dependent variable. In order to evaluate for an appropriate cutoff for screening potential reduction mammaplasty candidates, a receiver operating characteristic (ROC) curve was created to compare other relevant body metrics in addition to standard BMI (BMI^2.5^, BMI Prime, Corpulence Index, and Body Surface Area (BSA)). The area under the curve was compared to 0.5. All statistical analyses were performed with significance defined as *P*  < 0.05. All analysis was performed using SPSS (IBM, New York).

## Results

After screening the database, 1215 patients met inclusion criteria (Table 1). The average age at operation was 16.56 years. The vast majority of cases (1199, 98.69%) were performed in patients aged 14–18 years of age. There was no statistically significant association between age or age ranges and risk of complications. Overall, the average BMI was 30.7 kg/m^2^, and 593 (48.8%) were nonobese while 622 (51.2%) were obese. 28% were Class I, 15.6% were Class II, and 7.7% were Class III. Complications occurred in 18 (3%) of nonobese patients, versus 46 (7.4%) of obese patients, *P* < 0.05. The majority of patients in the sample were female (95.2%), while few identified as male or nonbinary (4.8%). The average operative time was 182.7 minutes (standard deviation (SD) = 72.1), and 166 (13.7%) of patients underwent an operation with a length greater than 1 SD above the mean. Most of the cohort were classified as American Society of Anesthesiologists (ASA) physical status of 1 (normal) (500, 41.2%) or 2 (mild systemic disease), and 78 (6.4%) were ASA 3 (severe systemic disease). Patients experiencing complications had a higher ASA physical status as well (*P* <0.05). Most patients had their surgery performed in an outpatient setting (939, 77.3%).

**Table 1:** Patient characteristics

	Overall Group		Complication Group	
	*n*	%	*n*	%
Overall	1215		64	5.27
Age, Mean (SD)	16.6 (1.35)		16.7 (0.981)	
Age, Range	9.95-18.0		13.7-17.9	
10–13	16	1.317	0	0
14–17	649	53.41	36	56.25
18	550	45.28	28	43.75
*Race*				
White	606	49.9	27	42.2
Unknown/Not Reported	186	15.3	9	14.1
Black or African-American	415	34.2	28	43.8
Asian	6	0.5	0	0.0
American Indian or Alaska Native	2	0.2	0	0.0
*Hispanic Identity*				
Hispanic	142	11.7	4	6.3
Non-Hispanic	1073	88.4	60	93.7
Gender				
Nonbinary	1	0.1	0	0.0
Male	57	4.7	1	1.6
Female	1157	95.2	63	98.4
Asthma History	86	7.1	7	10.9
*BMI*^*†*^				
Range	16.6–64.3		19.6–51.4	
<30	593	48.8	18	28.1
30-34.9 (Obese Class I)	340	28.0	15	23.4
35-39.9 (Obese Class II)	189	15.6	16	25.0
> 40 (Obese Class III)	93	7.7	15	23.4
Steroid Use	2	0.2	0	0.0
Hematologic Disorder	24	2.0	0	0.0
*Admission Status*				
Outpatient	939	77.3	47	73.4
Inpatient	276	22.7	17	26.6
*ASA Classification*^*†*^				
3	78	6.4	13	20.3
2	632	52.0	33	51.6
1	500	41.2	18	28.1
Operative Time, Mean (SD)	182.7 (72.1)		186.6 (67.6)	
Operative Time >1SD	166	13.7	9	14.1

†Indicates variable with significant association

A total of 64 (5.27%) of patients suffered from wound complications within 30 days. The most common complications were superficial wound disruption/dehiscence (44.93%) and superficial surgical site infection (42.03%). There was one incidence of mortality within 30 days of reduction mammaplasty in this cohort. The frequencies of the other surgical complications are displayed in Table 2.

**Table 2:** Complications

	*n*	% Of Complication Variable
	70	
*Wound Disruption/Dehiscence*		
Superficial	31	44.93
Deep	6	8.70
*Surgical Site Infection*		
Superficial	29	42.03
Deep	2	2.90
Organ/Space	1	1.45
Mortality*	1	

*Not included in complication variable

Following the multivariable analysis, three risk factors were found to have independent associations with complications (Table 3). These were having a BMI of 35–39.9 (Class II) (odds ratio [OR], 2.69; *P* = 0.007), a BMI  > 40 (Class III) (OR, 4.51; *P*  < .001), and an ASA class of 3 (OR, 2.57; *P* = 0.012). Other risk factors which did not reach statistical significance yet are otherwise notable include a BMI 30–34.9 (OR, 1.46), and a history of asthma (OR, 1.16).

**Table 3:** Independent risk factors following multivariable analysis

	Odds Ratio	*P* Value
*BMI*		
30-34.9 (Obese Class I)	1.46 [0.72, 2.95]	0.293
35-39.9 (Obese Class II)^†^	2.69 [1.31, 5.49]	0.007
>40 (Obese Class III)^†^	4.51 [2.03, 10.02]	<.001
ASA Class >3^†^	2.57 [1.24, 5.37]	0.012
Male Sex	0.41 [0.06, 3.10]	0.391
Non-White Race	0.99 [0.57, 1.72]	0.982
Hispanic Identity	0.53 [0.19, 1.53]	0.242
Asthma History	1.16 [0.48, 2.80]	0.743
Operative Time >1SD	0.99 [0.47, 2.10]	0.983
Outpatient Setting	0.81 [0.45, 1.46]	0.478

†Indicates variable with significant association

**Fig. 1:**
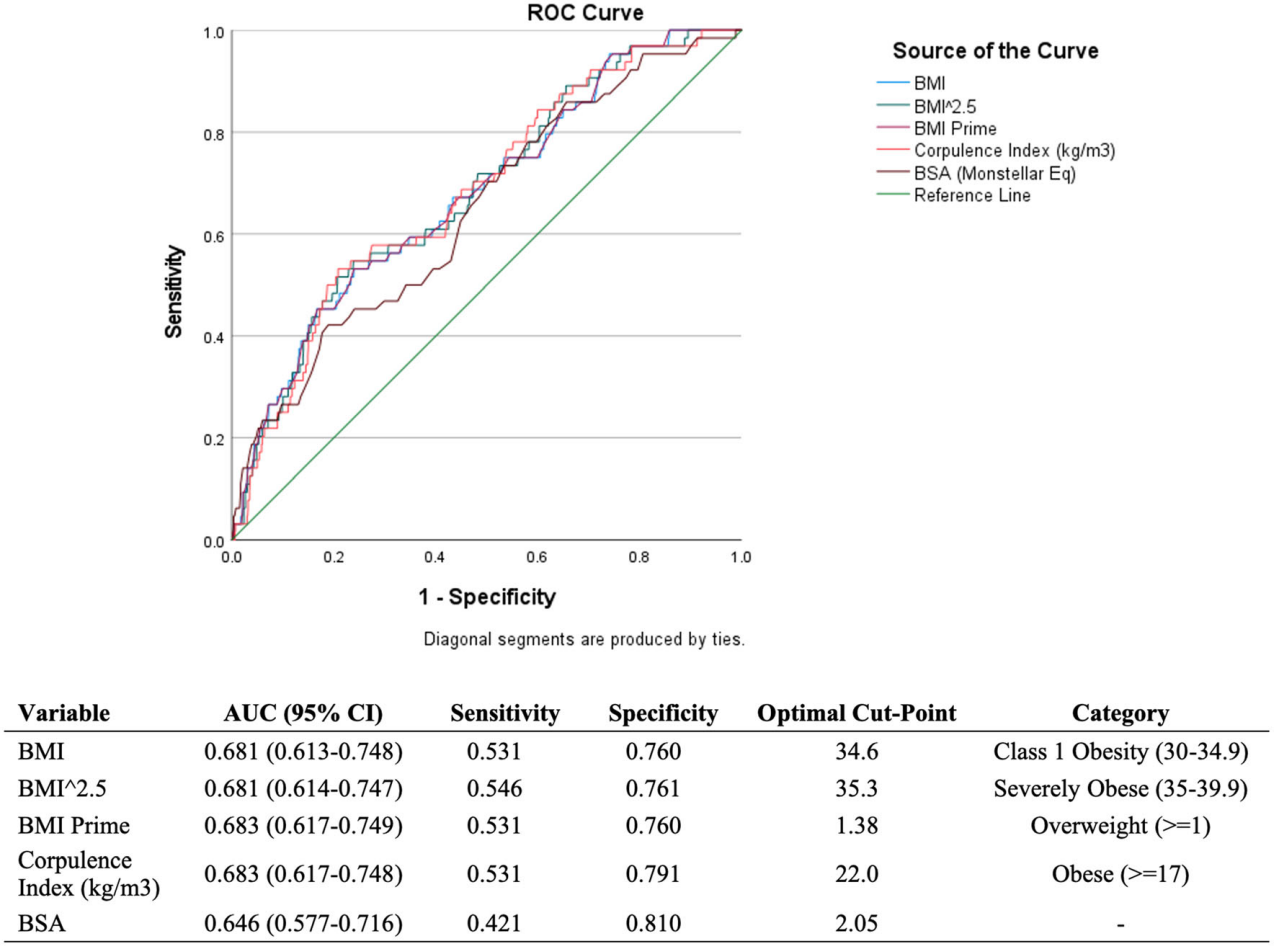
A ROC curve demonstrating that the area under the curve for BMI, among other metrics, was significantly different from 0.5, determining that an appropriate BMI cut point for increased wound complication risk in adolescent RM is 34.6. Other body metrics also had their own cut points

Because potential reduction mammaplasty patients present with a wide range of body mass indices, the ROC curve was created to further elucidate whether there was a potential BMI cut point to evaluate these patients and their risk for wound complications (Fig.1). Additionally, adding in other iterations and derivatives of BMI enables the ability to determine whether other uncommonly used metrics are better assessors of risk. The area under the curve (AUC) revealed potential cut points for all indices included (BMI, 34.6; BMI^2.5^, 35.3; BMI Prime, 1.38; Corpulence Index, 22.0 kg/m^3^; and BSA, 22.0).

## Discussion

This study leveraged the ACS NSQIP-P database to assess the overall rate of reduction mammaplasty in the adolescent population, with an overarching purpose of determining risk factors for wound-related complications in the short-term postoperative period. We hypothesized that adult risk factors such as obesity would also be reflected as risk factors in this adolescent population. The specific aims of the present study were to provide a broad update on the incidence of wound-related complications and determine whether any risk factors previously reported in the adult population would be seen in the adolescent population.

Our data revealed an overall low rate of complications, at 5.27%, which includes both superficial and deep wound disruption, dehiscence, and surgical site infections. WHO Class II/III obesity was demonstrated to be independent risk factors for these complications, specifically a BMI of 35–39.9 and higher. Contemporary literature has described reduction mammaplasty complication rates in all ages ranging from 7.1 to 53% [3, 9]. Among the few studies aimed at the adolescent population, the overall complication rates remain similar, ranging from 10 to 55%. The most frequently described complication include superficial wound dehiscence, surgical site infections, skin and/or fat necrosis, and hematoma [4, 5, 10]. These complications are similar to the ones studied here, with the most common complication being superficial wound disruption/dehiscence (44.93%) and superficial surgical site infections (42.03%) in our cohort.

Currently, the majority of the prior literature on this topic is comprised of single-center retrospective cohort studies, with results that align with ours; Kulkarni et al. reported a 10% incidence of wound dehiscence in their cohort of 60 adolescents undergoing reduction mammaplasty [8]. Because our investigation utilized predetermined variables from a third party, our complication incidence naturally trends toward lower complication rates compared to other studies using institutional data, which has the ability to include more granular data for other important complications such as fat necrosis and nipple–alveolar-related complications. However, our overall low complication rate remains close to the reported range and continues to demonstrate that reduction mammaplasty is a safe procedure for adolescents. As high BMI is strongly related to the presence of metabolic disorders, such as diabetes and a high lipid profile, these results are not surprising. Our ROC curve determined a potential BMI cut point of 34.6, which lies within the upper end of WHO Class I Obesity (30–34.9). This cut point is higher than our study cohort’s average BMI of 30.7. While other obesity indices also showed some potential utility as cut points, because BMI it is most commonly used and simplest to obtain, we believe that this should be adequate. These other cut points may be used at the surgeon’s discretion. Therefore, when considering breast reduction in obese patients, clinicians can consider using this objective cut point while remaining attentive to other important factors such as preoperative patient education, surgical planning, and postoperative management.

Our results also coincide with a similar study performed by Fairchild et al., which also utilized the NSQIP database, and reported an overall 7% complication rate in obese adolescents, which was defined as a BMI  ≥ 30, compared to 2% in nonobese adolescents. This study also reports 3.02 times higher odds of complications for obese adolescents. By stratifying obesity by WHO classification, we build upon their previous data by demonstrating a higher magnitude of complication odds with increasing BMI.

While the majority of publications delineating the relationship between obesity and reduction mammaplasty is centered around adults, these publications remain useful and can help guide our understanding of this association and its impact in adolescents. Nelson et al. also mined the adult NSQIP database and reported complications in adult (≥ 18 years) reduction mammaplasty patients [13]. Their study design is similar to ours, in which obesity is stratified by WHO classification, and their results also calculated a statistically significant higher rate of wound complications with increasing obesity classification, as high as 8.4% for Class III. The overall complication incidence in our study was 5.27%, which is comparable.

In analyses using different population-level data, previously reported results remain similar to ours. In 2011, Chen et al. utilized claims data from Blue Cross and Blue Shield to assess complications after different breast procedures in adults [14]. With regard to reduction mammaplasty, they found an 11.84 times higher adjusted odds ratio for any complication in obese patients compared to nonobese. However, this study was limited by their definition of obesity, allowing obesity to be diagnosed in a majority of cases by chart review, and not specifically as a BMI > 30. A systematic review examining the relationship between surgical complications and obesity following reduction mammaplasty by Myung et al. initially revealed mixed results between studies; however, following a pooled analysis, obese adults were shown to have a higher relative risk of surgical wound complications, including skin and fat necrosis [15]. The risk also increased with a higher BMI, reaching up to 2.05 odds for a BMI over 40.

Similarly, *Hudson et al.* also performed a systematic review to determine outcomes following breast reduction in the adolescent population specifically. Of the 2926 breast reductions included for analysis, 22.8% reported minor nonspecific postsurgical wound complications. And, while this review did not determine specific risk factors, we note that roughly one-third of the cases included were in adolescents of BMI >35. Included in that study’s outcome analysis was determination of breastfeeding outcomes. While surgical complications logically make up the majority of early complications, effects on breastfeeding are a long-term complication that certainly cannot be captured by databases limited to shorter window of time. Nelson et al. noted that 52.8% of the patients in their study did not attempt breastfeeding after having children, and of those who did attempt breastfeeding, only 55.1% were successful. However, an older systematic review performed by Thibaudeau et al. did not find a difference in breastfeeding after reduction mammaplasty within the first postpartum month [16]. At this time, no consensus has been reached on the exact relationship between reduction and breastfeeding, or whether any observed changes in breastfeeding ability are due to surgical or nonsurgical, i.e., psychological etiology. Breastfeeding following reduction should be an important topic to discuss with potential patients. We recommend further research into this potential complication.

Overall, obesity’s detrimental effect on wound healing is well known and well studied [17–19]. Obese patients suffer from chronic levels of low-grade inflammation, altered macrophage and myofibroblast function, and poorer collagen maturation, which all contribute to weak wound healing. Additionally obese patients are often concomitantly saddled with other chronic diseases such as diabetes and cardiovascular disease, which further compromise the wound healing process. The results shown in the current study, which are reinforced by previous publications on the subject, further emphasize the need for optimal patient selection and consideration of preoperative weight loss.

Furthermore, obesity also has the potential to affect multiple procedural aspects, leading to the wound complications capture in the present study. Obesity logically lends itself to longer operative times, as incisions and dissections must be lengthier and larger, thus having a cumulative effect on operative time. Larger retractors are typically used, and the increased weight of the tissue itself naturally causes more tension on the skin which can lead to necrosis, all contributing factors to wound problems. Additionally, the volume resected, which may skew higher in more obese patients, could have an impact on wound healing and complications. This has not been directly studied in adolescents, and the NSQIP-P is unable to track this variable, so further evaluation is needed to determine whether resection volume, irrespective of obesity, is a potential risk factor.

Difference in technique has also been a suggested reason for complications, and unfortunately, our study is limited by the inability to discern between different reduction mammaplasty techniques. With respect to surgical technique, as reported by Morrison et al. in their retrospective study of 80 adolescent reduction mammaplasty (160 breasts), there was no significant difference in complications between a medial pedicle Wise pattern versus medial pedicle short-scar incision [10]. We believe that our large sample likely generates a heterogeneous representation of reduction techniques. And because of this, we do not suspect that technique plays a significant confounding role in our results.

ASA physical status has also been a reported predictor of complications for multiple procedures. As an ASA class of ≥ 3 includes having a BMI  ≥ 40 by definition, it serves to reinforce the results seen with obesity alone in this study. In this study, ASA classification also helps to address the lack of comorbidity detail in the NSQIP-P, as higher ASA classification patients have more extensive comorbidities such as smoking, controlled/uncontrolled diabetes, hypertension, COPD, and other systemic problems. Based on these data, the presence of more and severe comorbidities does contribute to the risk of wound complications. We additionally suggest that further study is needed to determine which comorbidities or social factors, put adolescents at greater risk. And, although the overall complication rate in adolescents appears to be highly variable in the published literature, the overall trend points toward reduction mammaplasty being safer in younger patients, compared to adults. One proposed reason is that adolescents are generally healthier compared to adults, with years to decades less of comorbidity and metabolic burden [9, 10]. Therefore, early intervention for symptomatic macromastia may be a viable option for an overall healthier life in the future.

Despite the potential complications involved with reduction mammaplasty, its benefit is clear. In one study, 90% of patients were satisfied with their result, and 95% would have the procedure again [20]. Nguyen et al. performed a survey of women who underwent reduction mammaplasty for symptomatic macromastia at  < 21 years of age [21]. While this study was limited to women undergoing surgery between 1985 and 2005, with patient selection, surgical technique, and postoperative care likely less optimized and refined especially the earlier years included compared to contemporary methods, their results remain relevant to the current study. Both physiological symptoms including musculoskeletal pain and intertrigo improved alongside psychological and social problems, i.e., fitting in with peers, finding well-fitting clothes, and participation in extracurricular activities. However, decreased nipple sensation and difficulty breastfeeding were also reported. As previously described, these particular complications remain not well described and would ideally be assessed using long-term prospective cohort studies. As our study uses a large sample to help confirm the relationship between BMI and wound complications, future studies should aim to discuss other risk factors, or the mechanisms behind these risk factors. With further validation, BMI cutoffs could be developed to help risk-stratify this patient population. Finally, we suspect that wound complications could have a psychological impact on the patient’s perception of the final esthetic result, regardless of whether the complication truly affected the reduction’s appearance. Studies in adults have shown that complications can negatively affect the patient’s post-reduction satisfaction with their breasts, compared to other factors such as the incision pattern [22]. As breast reduction is relatively frequently sought out by adolescents, the use of validated patient reported outcome measurement tools such as the BREAST-Q would be a further area of interest.

## Limitations

There are limitations to consider when evaluating the results of our study. While administrative databases such as the ACS NSQIP-P are large and collect a wealth of data, their design allows for inherent reporting errors and sampling bias. Unlike retrospective studies using data mined from an institution’s own charts and records, the NSQIP datasets lack a level of granular detail. The absence of details including preoperative macromastia size, pedicle choice, blood supply, amount of tissue resected, variations in anesthesia, and postoperative management renders the authors unable to determine whether these factors are more strongly correlated with negative outcomes. One of the most feared and important complications following reduction is nipple areolar complex necrosis, which cannot be assessed by the level of detail provided by the NSQIP database. Similarly other common complications following breast surgery such as hematoma and seroma were also not able to be studied. Specific to the pediatric subset of NSQIP data, resident involvement is not documented, which has been shown to increase operative time across multiple surgical procedures; however, resident involvement’s actual effect on complication rates remains somewhat mixed [23–25]. Furthermore, this procedure is also commonly performed in private ambulatory surgery centers, which would not typically be tracked by this database. Additionally, 19-year-old patients, who fall under the WHO definition of adolescence are excluded by the database itself, limiting the power of the study. Future studies should aim to include this subset of patients. Finally, this database also does not collect surgeon or hospital case volume. In theory, more experienced surgeons and surgeons specializing in the pediatric surgery would yield better results.

## Conclusions

Our group’s retrospective study investigated the relationship between BMI and risk of short-term postoperative wound complications in adolescents undergoing reduction mammaplasty. We observed that higher WHO-classified BMI levels were associated with an independent compounding effect on the risk of postoperative wound complications, with a potential BMI cut point of assessing risk in this patient population at 34.6. We emphasize that wound complication rates in adolescent reduction mammaplasty remained low and should not be weighed heavily against the substantial benefits of the procedure. Therefore, our data can serve as information for preoperative counseling of adolescent patients and their families for patients that fall above this cut point, alongside other factors such as patient symptomatology, motivation, family support, and medical comorbidities. Moreover, it decreases the necessity of relying solely on adult reduction mammaplasty data for these evaluations.
